# Phenotype-Based Genetic Association Studies (PGAS)—Towards Understanding the Contribution of Common Genetic Variants to Schizophrenia Subphenotypes

**DOI:** 10.3390/genes5010097

**Published:** 2014-02-27

**Authors:** Hannelore Ehrenreich, Klaus-Armin Nave

**Affiliations:** 1 Max Planck Institute of Experimental Medicine, Hermann-Rein-Str.3, 37075 Göttingen, Germany; 2 DFG Center for Nanoscale Microscopy and Molecular Physiology of the Brain (CNMPB), Hermann-Rein-Str.3, 37075 Göttingen, Germany

**Keywords:** mental disease, diagnosis, classification systems, GWAS, monozygotic twins, deep phenotyping, Manhattan plot, genotype, SNP, GRAS data collection

## Abstract

Neuropsychiatric diseases ranging from schizophrenia to affective disorders and autism are heritable, highly complex and heterogeneous conditions, diagnosed purely clinically, with no supporting biomarkers or neuroimaging criteria. Relying on these “*umbrella diagnoses*”, genetic analyses, including genome-wide association studies (GWAS), were undertaken but failed to provide insight into the biological basis of these disorders. “Risk genotypes” of unknown significance with low odds ratios of mostly <1.2 were extracted and confirmed by including ever increasing numbers of individuals in large multicenter efforts. Facing these results, we have to hypothesize that thousands of genetic constellations in highly variable combinations with environmental co-factors can cause the individual disorder in the sense of a final common pathway. This would explain why the prevalence of mental diseases is so high and why mutations, including copy number variations, with a higher effect size than SNPs, constitute only a small part of variance. Elucidating the contribution of normal genetic variation to (disease) phenotypes, and so re-defining disease entities, will be extremely labor-intense but crucial. We have termed this approach PGAS (“phenotype-based genetic association studies”). Ultimate goal is the definition of biological subgroups of mental diseases. For that purpose, the GRAS (Göttingen Research Association for Schizophrenia) data collection was initiated in 2005. With >3000 phenotypical data points per patient, it comprises the world-wide largest currently available schizophrenia database (N > 1200), combining genome-wide SNP coverage and deep phenotyping under highly standardized conditions. First PGAS results on normal genetic variants, relevant for e.g., cognition or catatonia, demonstrated *proof-of-concept*. Presently, an autistic subphenotype of schizophrenia is being defined where an unfortunate accumulation of normal genotypes, so-called pro-autistic variants of synaptic genes, explains part of the phenotypical variance. Deep phenotyping and comprehensive clinical data sets, however, are expensive and it may take years before PGAS will complement conventional GWAS approaches in psychiatric genetics.

## 1. Schizophrenia Is a Heterogeneous Group of Diseases Diagnosed Purely Clinically

The diagnosis of schizophrenia (as of most mental diseases) is to this day an exclusively clinical one which, based on the leading classification systems, DSM and ICD, demands the simultaneous presence of a number of symptoms that are labeled “positive” or “negative”. In addition, persistence of these symptoms for at least 6 months is requested. As such, schizophrenia is the unifying term of a highly complex and heterogeneous group of multigenetic disorders, with an array of environmental hazards having influenced onset and course. Common to this group of disorders are merely some gross phenotypical traits. Biomarkers in unequivocal support of the diagnosis are missing and also modern imaging technologies, despite revealing brain matter loss or various functional alterations, have not yet assisted in better understanding disease etiology or pathogenesis, or in defining objective diagnostic criteria. This conglomerate of uncertainties, however, is the groundwork on which genetic studies have been and are being built on.

## 2. Despite High Heritability of Schizophrenia No “Disease Genes” Have Been Uncovered

There is no doubt that schizophrenia has a major genetic root. Family studies yielded heritability estimates of up to 80% for schizophrenia [1,2]. Monozygotic twins manifest concordance rates of ~50%, thus—on top of a clear genetic origin—pointing to a considerable influence of non-genetic causes, *i.e.*, environmental and/or epigenetic factors [3,4,5,6]. Searching for the genetic part of schizophrenia etiology, a considerable number of explorations have been undertaken, ranging from segregation or linkage analyses to association studies based on candidate genes [7,8]. Most of the so identified “disease genes”, e.g., *DISC1* [9], turned out not to respect disease borders. In fact, the *DISC1* translocation was found associated as much with mental health as with affective diseases, schizophrenia, autism, or personality disorders [10]. *DISC1* and others may thus at best deserve the label of a global risk gene for mental disorders.

So far, no definite “schizophrenia genes” of general significance could be identified. Recently reported genome-wide association studies (GWAS) of schizophrenia identified a number of genetic risk markers significantly associated with the disease, but unfortunately could not extract any universally convincing “disease genes”. The initially limited reproducibility over studies and ethnicities has improved with increasing numbers to ~60,000 individuals (PGC, Psychiatric Genetic Consortium) [11]. Associations, however, remain hampered by very low odds ratios (OR distinctly <1.2) [12,13]. Altogether, it appears highly unlikely that even larger GWAS, based on the umbrella term “schizophrenia”, will succeed in unraveling the genetic basis of these conditions or in identifying relevant disease genes.

3. “GWAS Hits” Do Not Predict Disease Severity or Other Relevant Schizophrenia Phenotypes

Which information do we then gain from the GWAS-identified risk genotypes? In fact, even an accumulation of the GWAS-identified “top 10” risk genotypes [14] does not lead to a more severe disease phenotype. In other words: If an individual possessed all “top 10” risk markers at the same time (“accumulated risk”), his disease will not be more grave than if he carried none of the risk genotypes.

Another attempt to make use of the GWAS-derived information has been the definition of polygenic schizophrenia risk scores (PSS). Besides the genome-wide significant risk loci, a substantial proportion of schizophrenia risk has been hypothesized to lie in markers not achieving genome-wide significance. Thus, quantitative PSS were calculated based on nominal associations of each SNP from the PGC GWAS. These PSS explained up to 6% of variance regarding the diagnosis schizophrenia in an independent sample [11]. Subsequently, PSS effects on various disease-relevant phenotypes, e.g., brain matter dimensions, were explored with variable success [15,16]. Employing the GRAS data collection of >1200 well characterized schizophrenic subjects (see below), we could not uncover the slightest association of PSS with any lead features of schizophrenia. In contrast, we found in the same schizophrenic population dramatic effects of accumulated environmental risk on age at onset of the disease [17].

## 4. Definition of Biological Subgroups of Schizophrenia: The Essential Next Step

**Figure 1:**
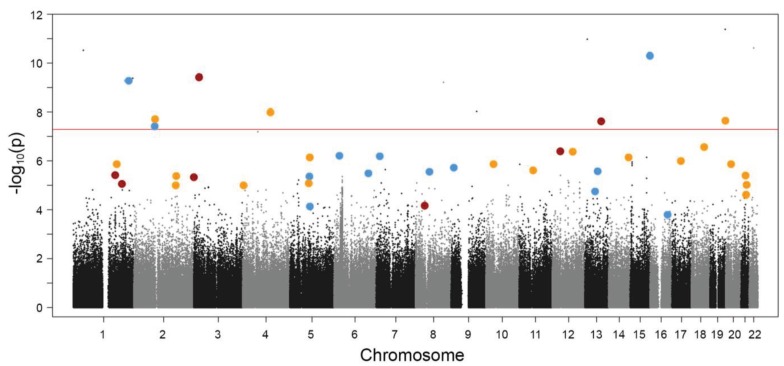
Schematic presentation of three hypothetical subphenotypes of schizophrenia embedded in a sample Manhattan plot. The non-overlapping red, blue or yellow dots comprise genetic constellations (“assemblies”) suggested to account for disease subforms with respective subphenotypes. Note that typical genome-wide association studies (GWAS) “top hits”, as defined by the highest significance levels for the clinical endpoint diagnosis (“schizophrenia”), most likely fall into different assemblies. This can explain their low odd ratios in a large and diverse patient group and the apparent lack of interactions. The depicted schema is purely hypothetical and shall illustrate the difficulties to define disease genes by conventional GWAS approaches based on endpoint diagnoses.

In summary, we note that all genetic “schizophrenia markers” discovered by GWAS up to date do not contribute more to the entirety of this disease than a minimally heightened probability (OR <1.2) of receiving the “umbrella diagnosis schizophrenia”. This whole picture could, however, look quite different when considering biological subgroups of schizophrenia as illustrated in Figure 1. GWAS on well-defined disease-relevant subphenotypes may ultimately not only lead to the identification of entirely new risk genotypes but, importantly, also to a re-distribution of some of the identified GWAS risk markers to certain subgroups, resulting then in much higher odds ratios.

In order to define biological subgroups of schizophrenia, the GRAS (*Göttingen Research Association for Schizophrenia*) data collection was initiated in 2004 [18,19]. The lead hypothesis of GRAS states that schizophrenia is caused by thousands of possible combinations of genetic markers interacting with a large array of different environmental risk factors. There may be rare cases where a ‘per se’ critical genetic load exists and the disease onset is independent of additional external factors. In the vast majority of cases, however, only the interaction between certain genetic predispositions and environmental factors will lead to disease onset. The overlap of genetic risk factors between individual schizophrenic patients/families probably is fairly low. This in turn might explain why it is obviously impossible to obtain common risk genes of schizophrenia with convincing odds ratios. According to the primary GRAS hypothesis, schizophrenia is the result of a combination of ‘unfortunate’ normal genetic variants exposed to unfavorable environmental influence. Apart from its scientific significance, undoubtedly, this hypothesis strongly supports any anti-stigma campaign.

We hypothesize that a considerable proportion of the population across cultures (likely ~50%) may harbor a principal genetic make-up for developing mental diseases whereas the remaining 50% of individuals could never acquire them due to absence of respective genetic prerequisites. Only in a small fraction of risk carriers the disease will break out (~0.5%–1% of the population across cultures) [20], co-induced by a multitude of potential external inputs, while the bulk of carriers will stay healthy and forward their predisposition to their offspring.

## 5. Deep Phenotyping Is a Prerequisite for PGAS: The GRAS Data Collection

If all human genetic approaches to schizophrenia as a “classic” genetic disease apparently failed, how will it be possible to learn more about the contribution of genes/genotypes to relevant disease subphenotypes? Motivated by this central question we started an alternative and at the same time complementary approach to conventional GWAS. We call this approach PGAS, which stands for *phenotype-based genetic association study*. PGAS enables us to explore the contribution of genes/genetic markers to schizophrenic subphenotypes. To start PGAS, we had to establish a comprehensive standardized phenotypical characterization of schizophrenic patients, the above introduced GRAS data collection. Within a few years we compiled the presently world-wide largest phenotypical database of schizophrenic patients comprising presently ~1200 subjects, with slow steady-state recruitment ongoing. Although much larger data bases of schizophrenic subjects exist, unfortunately, they do not even come close to the deep phenotyping information of GRAS. A total of 23 psychiatric hospitals all over Germany were involved as hosting centers in this effort [18,19]. Inclusion criteria for the patients were confirmed diagnosis of schizophrenia or schizoaffective disorder, and the ability to cooperate at least to a minimal degree. The GRAS population is representative for those schizophrenic patients in central Europe/Germany who are in contact with the healthcare system. Unrivalled in the GRAS data collection is the fact that all cross-sectional examinations of all patients were performed by one and the same travelling team. This fact contributes in a highly significant manner to the homogeneity of the resulting database, containing at present 82% schizophrenic and 18% schizoaffective subjects [18,19].

The GRAS data collection does not only encompass sociodemographic and basic clinical parameters but also, for example, a very comprehensive cognitive test battery, an extensive investigation of neurological signs and symptoms, a thorough medication history, several tests of extrapyramidal side effects of antipsychotic medication, complete information on drug abuse/addiction or other comorbidities, to name just a few of the covered data modules. Apart from the comprehensive cross-sectional analyses, we collected nearly all psychiatric medical reports and discharge letters of all GRAS patients, allowing also for fairly solid longitudinal data analysis, e.g., information on disease course and outcome. Altogether, we have about 3000 data points per patient, which allow us to perform PGAS [18,19]. Importantly, most GRAS patients agreed to be re-contacted for follow-up studies, thereby enabling targeted analyses in the future, e.g., detailed morphometrical or functional magnetic resonance imaging studies, or inducible pluripotent stem cell approaches to study cellular consequences of complex genotypes.

## 6. Phenotype-Based Genetic Association Studies (PGAS): Proof-of-Principle

First *proof-of-principle* studies for the PGAS approach were performed over the last years. We could show that genetic variants within the *complexin2* gene (6 single nucleotide polymorphisms) influence the cognitive capability of schizophrenic patients [18]. This gene encodes a synaptic protein that is crucial for the regulation of neurotransmitter release. Similarly, the calcium-activated potassium channel SK3 is involved in the modulation of neuroplasticity and influences cognition of schizophrenic individuals genotype-dependently (as a function of the length of a CAG repeat in the *N*-terminal region) [21]. Moreover, a distinct combination of erythropoietin (EPO) and EPO receptor (EPOR) genotypes leads to a remarkable cognitive benefit in schizophrenic subjects as compared to all other possible EPO/EPOR genotype combinations [22]. Of note, the association of certain genetic variants with cognition in schizophrenia may likewise hold true for healthy individuals and other disease populations [22]. This clearly emphasizes the importance of investigating the genotype contribution to phenotypes in general, rather than immediately focusing on risk genes in complex diseases.

Among the discussed candidate risk genes of schizophrenia is *NRG1* [23,24,25]. Analyzing its disease-relevance in the GRAS population yielded for the first time an association with age at disease onset and severity of positive symptoms [26]. A myelin-associated gene, *CNP*, turned out to co-determine the occurrence of a catatonia-depression syndrome upon aging [27]. Importantly, in most of these PGAS approaches, either replication in an independent human cohort and/or behavioral phenotypes in corresponding mouse mutants were demonstrated. In fact, the translation to mouse models, and even to cultured cells, plays an important part in the search for mechanistic comprehension of the observed phenomena.

## 7. PGAS Will Be Instrumental for Definition of Biological Schizophrenia Subgroups

Even though the above mentioned first *proof-of-principle* studies were important to set the stage for PGAS, it is now mandatory to work on the “bigger picture”. The availability of genome-wide genetic data on the GRAS sample enables us to better understand the genetic basis of neuropsychiatric phenotypes. Using the Axiom^TM^ Caucasian European array as a backbone, we have enriched it by including additional markers of putative functional importance [28]. At present, we are in the process of defining biological subphenotypes of schizophrenia which are less complex as compared to the ‘umbrella diagnosis schizophrenia’ and can be described and well quantified by respective phenotype scores. Prerequisite for applying these scores to genetic studies is a high internal consistency of the selected score items, reflected by a demanded Cronbach’s alpha of >80%. As a first example, we have operationalized an autistic subphenotype of schizophrenia. This subphenotype, which comprises lead features of high-functioning autism (deficits in social interaction and communication, as well as repetitive behaviors/stereotypies), shows a normal distribution in the GRAS population, thus allowing for extreme group comparisons with respect to the associated genotypical information. Our primary target genes for the autistic subphenotype are synaptic genes where we find an unfortunate accumulation of normal genotypes, that we consider as “pro-autistic” variants of these genes, to explain an appreciable part of the phenotypical variance. A sample of Asperger autists is presently recruited with the aim to replicate the genotype-phenotype associations found in the autistic subgroup of schizophrenic individuals. Similarly, a psychomotor and a schizoaffective subphenotype of schizophrenia will be phenotypically as well as—subsequently—genetically characterized, targeting myelin- and ion channel-associated genes, respectively [29].

## 8. Conclusions

To conclude, the lack of objective diagnoses and the non-existence of classical disease genes have forced us to re-consider the genetic approach to mental diseases. Deep clinical phenotyping as prerequisite for PGAS, combined with genome-wide SNP coverage, makes it possible to improve our understanding of the molecular-genetic architecture of schizophrenia and likely other mental diseases via systematic phenotype-based approaches. These in turn necessitate highly labor-intense groundwork since they depend on the availability of comprehensive phenotypical databases comparable to the GRAS data collection. Some efforts in a similar direction are launched in other disciplines, for instance in a first trial to integrate available electronic medical record data and GWAS information [30]. Regarding the depth of phenotyping, however, this approach certainly needs to be substantially improved. Even if “deep phenotyping” is much more tedious than collecting thousands of subjects with merely a certain diagnostic label versus healthy individuals, it will be the only way to reach the goal, *i.e.*, novel definitions of biologically sound disease subgroups. It can only be hoped that big consortia and sponsors will follow this rationale such that the number of deeply phenotyped individuals will grow. At the end of the road, PGAS will permit better insights into the complex genotype-phenotype interactions of schizophrenia (and other neuropsychiatric diseases) and in this way open up more targeted therapeutic strategies for biological subphenotypes of the disease.
